# Crystal structure of bis­[*N*-phenyl-2-(1,2,3,4-tetrahydronaphthalen-1-ylidene)hydrazinecarbothio­amidato-κ^2^ *N* ^2^,*S*]zinc dimethyl sulfoxide monosolvate

**DOI:** 10.1107/S205698901500612X

**Published:** 2015-04-02

**Authors:** Genelane Cruz Santana, Iara de Fátima Gimenez, Christian Näther, Inke Jess, Adriano Bof de Oliveira

**Affiliations:** a Programa de Pós-Graduação em Ciência e Engenharia de Materiais, Universidade Federal de Sergipe, Av. Marechal Rondon s/n, 49100-000 São Cristóvão-SE, Brazil; b Institut für Anorganische Chemie, Christian-Albrechts-Universität zu Kiel, Max-Eyth Strasse 2, D-24118 Kiel, Germany; c Departamento de Química, Universidade Federal de Sergipe, Av. Marechal Rondon s/n, 49100-000 São Cristóvão-SE, Brazil

**Keywords:** crystal structure, thio­semi­carba­zone–Zn^II^ complex, hydrogen-bonded polymer

## Abstract

The reaction of the *N*-phenyl-2-(1,2,3,4-tetrahydronaphthalen-1-yl­idene)hy­dra­zine­car­bo­thio­amide ligand with zinc acetate dihydrate in a 2:1 molar ratio yielded a yellow solid, which was crystallized from DMSO to obtain the title compound, [Zn(C_17_H_16_N_3_S)_2_]·C_2_H_6_OS. The Zn^II^ ion is four-coordinated in a distorted tetra­hedral environment by two deprotonated ligands. Each ligand acts as an *N*,*S*-donor, forming a five-membered metallacycle. The maximum deviation from the mean plane of the N–N–C–S chelate group is 0.0029 (14) Å for the *N*-donor atom of one ligand and 0.0044 (14) Å for the non-coordinating N atom of the second. The dihedral angle between the planes of the two chelate groups is 72.80 (07)°. Bond lengths in the ligands are compared with those in the crystal structure of the free ligand. In the crystal, complex mol­ecules are connected by dimethyl sulfoxide solvate mol­ecules *via* N—H⋯O hydrogen-bonding inter­actions, building a one-dimensional hydrogen-bonded polymer along the *a*-axis direction. The S atom and one C atom of the dimethyl sulfoxide solvate mol­ecules are disordered over two sets of sites with an occupancy ratio of 0.6:0.4.

## Abstract 2

The synthesis and crystal structure of a Zn^II^ complex with a (3,4-di­hydro­naphthalen-1(2*H*-yl­idene)-*N*-phenyl-carbamohydrazino­thio­ate ligand is reported. The crystal structure shows DMSO mol­ecules bridging the complex units, building an one-dimensional H-bonded polymer.

## Chemical context   

In a continuation of our on-going research on the supra­molecular chemistry of thio­semicarbazone derivatives and their complexes, we report herein the synthesis and crystal structure of a Zn^II^ complex with the *N*-phenyl-2-(1,2,3,4-tetrahydronaphthalen-1-yl­idene)hy­dra­zine­car­bo­thio­amidate ligand. Thio­semicarbazone derivatives are *N*,*S*-donors with a wide range of coordination modes and a variety of applications in biological inorganic chemistry (Lobana *et al.* 2009 ▸; Ferraz *et al.* 2012 ▸).
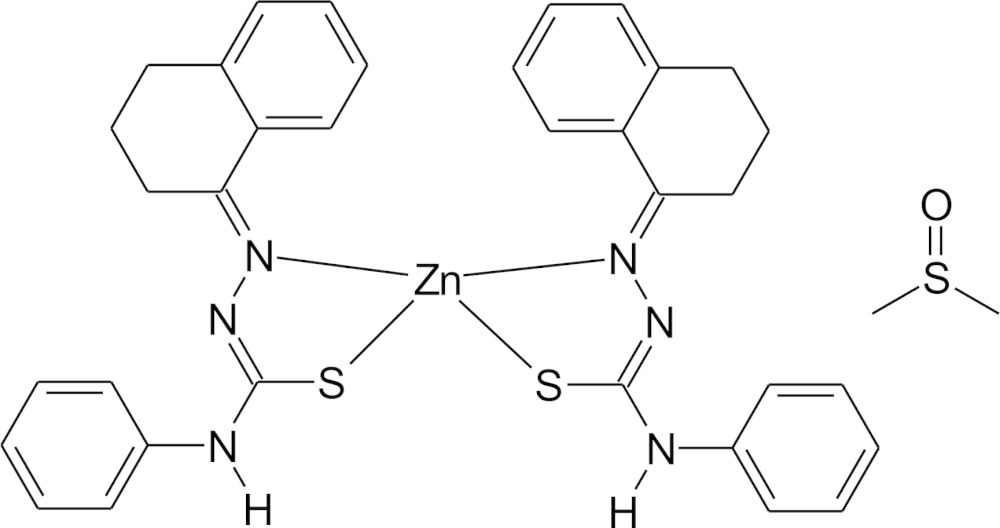


## Structural commentary   

**Figure 1:**
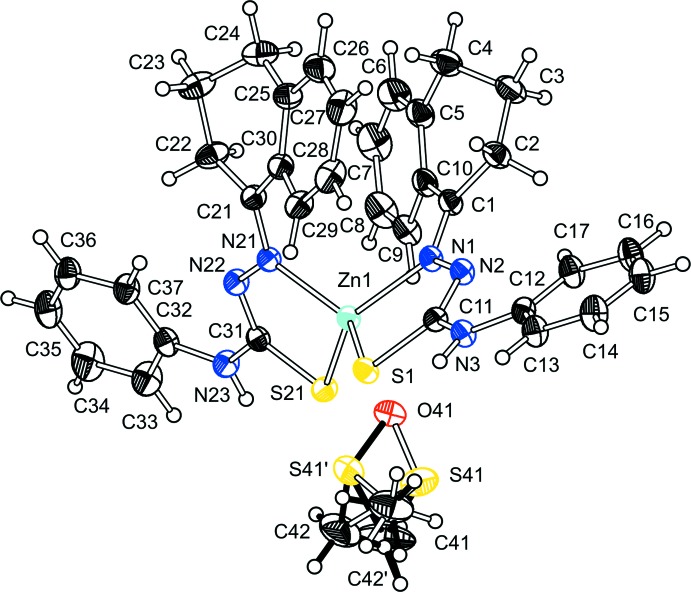
The mol­ecular structure of the title compound with atom labeling and displacement ellipsoids drawn at the 30% probability level. Disorder is shown with open and full bonds.

The mol­ecular structure of the title compound consists of one Zn^II^ ion, four-coordinated in a distorted tetra­hedral environment by two deprotonated thio­semicarbazone ligands in a bidentate chelating mode, and one disordered DMSO solvate mol­ecule (Fig. 1 ▸). The *N*,*S*-donor atoms together with the central zinc atom form five-membered metallacycles (Fig. 1 ▸). The maximum deviation from the mean plane of the N1—N2—C11—S1 chelate group is 0.0029 (14) Å for the N1 donor atom. For the N21—N22—C31—S21 chelate group, the maximum deviation is 0.0044 (14) Å for atom N22. The dihedral angle between the planes of the two chelate groups is 72.80 (7)°, clearly showing the distorted tetra­hedral geometry.

The acidic hydrogen of the hydrazine fragment is lost by the reaction with the acetate anion. The negative charge of the deprotonated ligand is delocalized over the N—N—C—S entity, as indicated by their inter­mediate bond lengths. The bond lengths in the ligand are also affected by the coordination with the metal atom, especially the C—S bond length, which is consistent with increased single-bond character. In the crystal structure of the free ligand (de Oliveira *et al.*, 2014 ▸), selected bond lengths are N—N = 1.3846 (14), N—C = 1.3642 (16) and C—S = 1.6773 (13) Å. For the ligands in the title Zn^II^ complex, the bond lengths are N1—N2 = 1.400 (3)/N21—N22 = 1.393 (3) Å, N2—C11 = 1.303 (3)/N22—C31 = 1.304 (3) Å and C11—S1 = 1.755 (2)/C31—S21 = 1.749 (2) Å.

Neither of the coordinating ligands is planar. For one ligand, the dihedral angles between the aromatic rings (C5–C10 and C12–C17) is 58.25 (11)°. In the second ligand, the corresponding angle is 49.99 (11)° between the C25–C30 and C32–C37 rings. In addition, the aliphatic rings are also not planar. The maximum deviation from the mean plane for the C1–C5/C10 ring is 0.355 (3) Å for C3 and for the C21–C25/C30 ring the maximum deviation is 0.359 (3) Å for C23, with both of the aliphatic rings having an envelope conformation

## Supra­molecular features   

**Figure 2:**
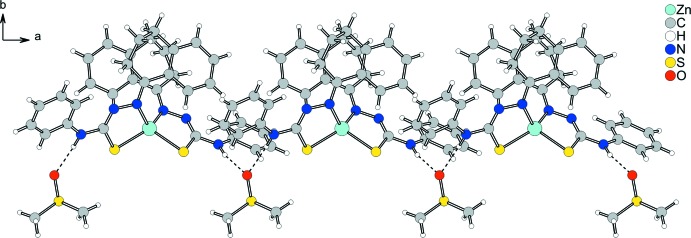
View of the one-dimensional hydrogen-bonded polymer that elongates along the *a*-axis direction. Inter­molecular hydrogen bonding (for details, see Table 1 ▸) is shown as dashed lines. The minor occupancy components of the disordered atoms are not shown for clarity.

In the crystal, the Zn^II^ complex mol­ecules and the DMSO solvent mol­ecules build a monomeric entity. The DMSO mol­ecule bridges two complex mol­ecules *via* inter­molecular N—H⋯O hydrogen-bonding inter­actions, building a one-dimensional hydrogen-bonded polymer along the *a*-axis direction (Fig. 2 ▸, Table 1 ▸).

## Synthesis and crystallization   

Starting materials were commercially available and used without further purification. The ligand synthesis was adapted from a procedure reported previously (Freund & Schander, 1902 ▸). A mixture of *N*-phenyl-2-(1,2,3,4-tetra­hydro­naph­tha­len-1-yl­idene)hy­dra­zine­car­bo­thio­amide dissolved in THF (2 mmol/40 mL) with zinc acetate dihydrate dissolved in ethanol (1 mmol/30 mL) was refluxed for 4 h under continuous stirring. An orange solid was obtained, filtered and washed with ethanol. Suitable crystals for X-ray diffraction were obtained in DMSO by slow evaporation of the solvent.

## Refinement   

Crystal data, data collection and structure refinement details are summarized in Table 2 ▸. The C—H and N—H hydrogen atoms were positioned with idealized geometry and refined isotropically with *U*_iso_(H) = 1.2 *U*_eq_(C) (1.5 for methyl H atoms) using a riding model with C—H = 0.95 Å for aromatic, C—H = 0.99 Å for methyl­ene, C—H = 0.98 Å for methyl and N—H = 0.88 Å. In the DMSO solvate mol­ecule, the S atom and methyl­ene C atom C42 and attached H atoms are disordered and were refined using a split model with an occupancy ratio of 0.4:0.6.

## Supplementary Material

Crystal structure: contains datablock(s) I, publication_text. DOI: 10.1107/S205698901500612X/im2462sup1.cif

Structure factors: contains datablock(s) I. DOI: 10.1107/S205698901500612X/im2462Isup2.hkl

CCDC reference: 1056177

Additional supporting information:  crystallographic information; 3D view; checkCIF report

## Figures and Tables

**Table 1:** Hydrogen-bond geometry (, )

*D*H*A*	*D*H	H*A*	*D* *A*	*D*H*A*
N3H3*N*O41	0.88	2.08	2.945(3)	168
N23H23*N*O41^i^	0.88	2.03	2.903(3)	173

Symmetry code: (i) 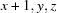.

**Table 2:** Experimental details

Crystal data	
Chemical formula	[Zn(C_17_H_16_N_3_S)_2_]C_2_H_6_OS
*M*_r_	732.27
Crystal system, space group	Monoclinic, *P*2_1_/*n*
Temperature (K)	200
*a*, *b*, *c* ()	10.6320(4), 17.2695(5), 19.4067(7)
()	94.223(3)
*V* (^3^)	3553.6(2)
*Z*	4
Radiation type	Mo *K*
(mm^1^)	0.91
Crystal size (mm)	0.14 0.10 0.06

Data collection	
Diffractometer	STOE IPDS1
Absorption correction	Numerical (*X-SHAPE* and *X-RED32*; Stoe Cie, 2008 ▸)
*T*_min_, *T*_max_	0.793, 0.916
No. of measured, independent and observed [*I* > 2(*I*)] reflections	45253, 6947, 6171
*R*_int_	0.041
(sin /)_max_ (^1^)	0.617

Refinement	
*R*[*F* ^2^ > 2(*F* ^2^)], *wR*(*F* ^2^), *S*	0.042, 0.097, 1.06
No. of reflections	6947
No. of parameters	442
H-atom treatment	H-atom parameters constrained
_max_, _min_ (e ^3^)	0.80, 0.68

Computer programs: *X-AREA* and *X-RED32* (Stoe Cie, 2008 ▸), *SHELXS97* (Sheldrick, 2008 ▸), *SHELXL2013-2* (Sheldrick, 2015 ▸), *DIAMOND* (Brandenburg, 2006 ▸) and *publCIF* (Westrip, 2010 ▸).

## supplementary crystallographic information

### Crystal data

**Table:**

[Zn(C_17_H_16_N_3_S)_2_]·C_2_H_6_OS	*F*(000) = 1528
*M**_r_* = 732.27	*D*_x_ = 1.369 Mg m^−^^3^
Monoclinic, *P*2_1_/*n*	Mo *K*α radiation, λ = 0.71073 Å
*a* = 10.6320 (4) Å	θ = 1.6–26.0°
*b* = 17.2695 (5) Å	µ = 0.91 mm^−^^1^
*c* = 19.4067 (7) Å	*T* = 200 K
β = 94.223 (3)°	Prism, orange
*V* = 3553.6 (2) Å^3^	0.14 × 0.10 × 0.06 mm
*Z* = 4	

### Data collection

**Table:**

STOE IPDS-1 diffractometer	6947 independent reflections
Radiation source: fine-focus sealed tube, STOE IPDS-1	6171 reflections with *I* > 2σ(*I*)
Graphite monochromator	*R*_int_ = 0.041
φ scans	θ_max_ = 26.0°, θ_min_ = 1.6°
Absorption correction: numerical (*X-SHAPE* and *X-RED32*; Stoe & Cie, 2008)	*h* = −13→13
*T*_min_ = 0.793, *T*_max_ = 0.916	*k* = −21→21
45253 measured reflections	*l* = −23→23

### Refinement

**Table:**

Refinement on *F*^2^	Primary atom site location: structure-invariant direct methods
Least-squares matrix: full	Secondary atom site location: difference Fourier map
*R*[*F*^2^ > 2σ(*F*^2^)] = 0.042	Hydrogen site location: inferred from neighbouring sites
*wR*(*F*^2^) = 0.097	H-atom parameters constrained
*S* = 1.06	*w* = 1/[σ^2^(*F*_o_^2^) + (0.0382*P*)^2^ + 2.9458*P*] where *P* = (*F*_o_^2^ + 2*F*_c_^2^)/3
6947 reflections	(Δ/σ)_max_ = 0.001
442 parameters	Δρ_max_ = 0.80 e Å^−^^3^
0 restraints	Δρ_min_ = −0.68 e Å^−^^3^

### Special details

**Table:**

Geometry. All e.s.d.'s (except the e.s.d. in the dihedral angle between two l.s. planes) are estimated using the full covariance matrix. The cell e.s.d.'s are taken into account individually in the estimation of e.s.d.'s in distances, angles and torsion angles; correlations between e.s.d.'s in cell parameters are only used when they are defined by crystal symmetry. An approximate (isotropic) treatment of cell e.s.d.'s is used for estimating e.s.d.'s involving l.s. planes.
Refinement. Refinement of *F*^2^ against ALL reflections. The weighted *R*-factor *wR* and goodness of fit *S* are based on *F*^2^, conventional *R*-factors *R* are based on *F*, with *F* set to zero for negative *F*^2^. The threshold expression of *F*^2^ > σ(*F*^2^) is used only for calculating *R*-factors(gt) *etc*. and is not relevant to the choice of reflections for refinement. *R*-factors based on *F*^2^ are statistically about twice as large as those based on *F*, and *R*- factors based on ALL data will be even larger.

### Fractional atomic coordinates and isotropic or equivalent isotropic displacement parameters (Å^2^)

**Table:**

	*x*	*y*	*z*	*U*_iso_*/*U*_eq_	Occ. (<1)
Zn1	0.75764 (2)	0.193873 (16)	0.627860 (14)	0.03590 (9)	
C1	0.7188 (2)	0.31745 (15)	0.73748 (13)	0.0415 (5)	
C2	0.6288 (3)	0.36245 (17)	0.77834 (15)	0.0518 (7)	
H2A	0.6079	0.3311	0.8186	0.062*	
H2B	0.5497	0.3711	0.7491	0.062*	
C3	0.6803 (3)	0.44078 (18)	0.80402 (17)	0.0635 (8)	
H3A	0.6101	0.4736	0.8177	0.076*	
H3B	0.7399	0.4331	0.8451	0.076*	
C4	0.7473 (3)	0.48091 (18)	0.74761 (18)	0.0633 (8)	
H4A	0.6883	0.4883	0.7062	0.076*	
H4B	0.7777	0.5324	0.7638	0.076*	
C5	0.8570 (3)	0.43171 (18)	0.72969 (15)	0.0555 (7)	
C6	0.9733 (3)	0.4637 (2)	0.71835 (19)	0.0716 (10)	
H6	0.9831	0.5184	0.7190	0.086*	
C7	1.0752 (3)	0.4175 (2)	0.7061 (2)	0.0784 (11)	
H7	1.1535	0.4406	0.6973	0.094*	
C8	1.0638 (3)	0.3377 (2)	0.70660 (18)	0.0684 (9)	
H8	1.1345	0.3059	0.6994	0.082*	
C9	0.9483 (3)	0.30455 (18)	0.71767 (15)	0.0524 (7)	
H9	0.9404	0.2498	0.7186	0.063*	
C10	0.8434 (2)	0.35090 (16)	0.72747 (14)	0.0467 (6)	
N1	0.68571 (18)	0.25148 (12)	0.70903 (10)	0.0374 (4)	
N2	0.56703 (18)	0.22579 (12)	0.72604 (10)	0.0390 (4)	
C11	0.5176 (2)	0.16915 (14)	0.68884 (12)	0.0365 (5)	
S1	0.58047 (6)	0.11959 (4)	0.62029 (3)	0.04070 (15)	
N3	0.40074 (18)	0.14264 (13)	0.70263 (11)	0.0407 (5)	
H3N	0.3667	0.1097	0.6721	0.049*	
C12	0.3262 (2)	0.15955 (15)	0.75765 (13)	0.0399 (5)	
C13	0.2066 (2)	0.12656 (17)	0.75371 (14)	0.0493 (6)	
H13	0.1790	0.0964	0.7146	0.059*	
C14	0.1274 (3)	0.1373 (2)	0.80637 (16)	0.0582 (8)	
H14	0.0455	0.1150	0.8027	0.070*	
C15	0.1657 (3)	0.1799 (2)	0.86376 (15)	0.0580 (8)	
H15	0.1117	0.1865	0.9002	0.070*	
C16	0.2836 (3)	0.2128 (2)	0.86770 (15)	0.0577 (8)	
H16	0.3107	0.2425	0.9073	0.069*	
C17	0.3644 (2)	0.20369 (18)	0.81488 (14)	0.0508 (7)	
H17	0.4450	0.2276	0.8182	0.061*	
C21	0.7844 (2)	0.34196 (15)	0.54269 (13)	0.0408 (5)	
C22	0.8717 (3)	0.39974 (16)	0.51388 (18)	0.0555 (7)	
H22A	0.9047	0.3781	0.4716	0.067*	
H22B	0.9442	0.4084	0.5480	0.067*	
C23	0.8086 (3)	0.47694 (18)	0.4965 (2)	0.0655 (9)	
H23A	0.8736	0.5160	0.4876	0.079*	
H23B	0.7518	0.4715	0.4540	0.079*	
C24	0.7336 (3)	0.50397 (18)	0.55528 (19)	0.0675 (9)	
H24A	0.7906	0.5108	0.5975	0.081*	
H24B	0.6939	0.5546	0.5434	0.081*	
C25	0.6338 (3)	0.44571 (17)	0.56840 (15)	0.0532 (7)	
C26	0.5159 (3)	0.4675 (2)	0.58907 (18)	0.0674 (9)	
H26	0.4992	0.5206	0.5971	0.081*	
C27	0.4235 (3)	0.4135 (2)	0.59806 (18)	0.0718 (10)	
H27	0.3456	0.4293	0.6146	0.086*	
C28	0.4430 (3)	0.3370 (2)	0.58337 (17)	0.0627 (8)	
H28	0.3776	0.3002	0.5875	0.075*	
C29	0.5585 (2)	0.31397 (17)	0.56250 (15)	0.0495 (6)	
H29	0.5712	0.2612	0.5509	0.059*	
C30	0.6571 (2)	0.36679 (16)	0.55815 (14)	0.0440 (6)	
N21	0.82246 (17)	0.27193 (12)	0.55813 (10)	0.0365 (4)	
N22	0.94220 (18)	0.25641 (12)	0.53682 (11)	0.0402 (5)	
C31	0.9966 (2)	0.19343 (14)	0.56117 (12)	0.0370 (5)	
S21	0.93916 (6)	0.12624 (4)	0.61853 (3)	0.04147 (15)	
N23	1.11279 (19)	0.17498 (13)	0.53992 (11)	0.0438 (5)	
H23N	1.1506	0.1358	0.5618	0.053*	
C32	1.1814 (2)	0.20924 (16)	0.48819 (13)	0.0434 (6)	
C33	1.2983 (3)	0.1765 (2)	0.47906 (17)	0.0627 (8)	
H33	1.3285	0.1346	0.5073	0.075*	
C34	1.3711 (3)	0.2057 (2)	0.4282 (2)	0.0763 (11)	
H34	1.4511	0.1833	0.4222	0.092*	
C35	1.3300 (3)	0.2660 (2)	0.38675 (17)	0.0636 (8)	
H35	1.3804	0.2853	0.3521	0.076*	
C36	1.2150 (3)	0.29789 (19)	0.39610 (15)	0.0551 (7)	
H36	1.1853	0.3396	0.3674	0.066*	
C37	1.1408 (3)	0.27054 (18)	0.44670 (14)	0.0499 (6)	
H37	1.0616	0.2941	0.4528	0.060*	
S41	0.22794 (16)	−0.03767 (8)	0.62486 (9)	0.0732 (4)	0.60
S41'	0.2187 (2)	−0.01998 (12)	0.55185 (11)	0.0653 (5)	0.40
O41	0.25110 (17)	0.04499 (11)	0.60282 (11)	0.0521 (5)	
C41	0.1008 (4)	−0.0730 (2)	0.5786 (3)	0.0915 (13)	
H41A	0.0241	−0.0510	0.5958	0.137*	0.60
H41B	0.0990	−0.1295	0.5831	0.137*	0.60
H41C	0.1051	−0.0591	0.5298	0.137*	0.60
H41D	0.0243	−0.0413	0.5780	0.137*	0.40
H41E	0.1230	−0.0914	0.6257	0.137*	0.40
H41F	0.0857	−0.1175	0.5477	0.137*	0.40
C42	0.3516 (9)	−0.0914 (6)	0.5996 (7)	0.124 (5)	0.60
H42A	0.4297	−0.0742	0.6249	0.186*	0.60
H42B	0.3586	−0.0846	0.5499	0.186*	0.60
H42C	0.3372	−0.1462	0.6095	0.186*	0.60
C42'	0.3297 (14)	−0.0806 (9)	0.5512 (7)	0.099 (5)	0.40
H42D	0.4055	−0.0550	0.5366	0.148*	0.40
H42E	0.3053	−0.1226	0.5190	0.148*	0.40
H42F	0.3471	−0.1019	0.5978	0.148*	0.40

### Atomic displacement parameters (Å^2^)

**Table:**

	*U*^11^	*U*^22^	*U*^33^	*U*^12^	*U*^13^	*U*^23^
Zn1	0.03107 (14)	0.03978 (16)	0.03778 (15)	0.00053 (11)	0.00889 (10)	−0.00104 (11)
C1	0.0421 (13)	0.0436 (14)	0.0397 (13)	−0.0017 (11)	0.0089 (10)	−0.0057 (11)
C2	0.0520 (15)	0.0492 (15)	0.0560 (17)	−0.0006 (13)	0.0169 (13)	−0.0114 (13)
C3	0.075 (2)	0.0528 (17)	0.0647 (19)	−0.0011 (15)	0.0165 (16)	−0.0208 (15)
C4	0.074 (2)	0.0467 (16)	0.070 (2)	−0.0102 (15)	0.0093 (16)	−0.0144 (14)
C5	0.0604 (17)	0.0546 (17)	0.0519 (16)	−0.0159 (14)	0.0073 (13)	−0.0132 (13)
C6	0.074 (2)	0.065 (2)	0.077 (2)	−0.0324 (18)	0.0117 (18)	−0.0151 (17)
C7	0.059 (2)	0.093 (3)	0.084 (3)	−0.037 (2)	0.0168 (18)	−0.018 (2)
C8	0.0451 (16)	0.088 (2)	0.073 (2)	−0.0164 (16)	0.0134 (15)	−0.0212 (19)
C9	0.0423 (14)	0.0620 (18)	0.0538 (16)	−0.0092 (13)	0.0091 (12)	−0.0149 (14)
C10	0.0465 (14)	0.0509 (15)	0.0436 (14)	−0.0117 (12)	0.0088 (11)	−0.0099 (12)
N1	0.0317 (10)	0.0431 (11)	0.0382 (11)	−0.0032 (8)	0.0080 (8)	−0.0028 (9)
N2	0.0320 (10)	0.0451 (11)	0.0409 (11)	−0.0039 (9)	0.0105 (8)	−0.0023 (9)
C11	0.0306 (11)	0.0390 (12)	0.0403 (13)	0.0008 (9)	0.0056 (9)	0.0017 (10)
S1	0.0355 (3)	0.0431 (3)	0.0445 (3)	−0.0037 (2)	0.0101 (2)	−0.0067 (3)
N3	0.0321 (10)	0.0481 (12)	0.0426 (11)	−0.0056 (9)	0.0077 (8)	−0.0052 (9)
C12	0.0324 (12)	0.0481 (14)	0.0401 (13)	0.0029 (10)	0.0077 (10)	0.0038 (11)
C13	0.0359 (13)	0.0656 (18)	0.0471 (15)	−0.0077 (12)	0.0076 (11)	−0.0030 (13)
C14	0.0334 (13)	0.086 (2)	0.0565 (17)	−0.0080 (14)	0.0122 (12)	0.0018 (16)
C15	0.0414 (14)	0.085 (2)	0.0495 (16)	0.0000 (14)	0.0177 (12)	−0.0010 (15)
C16	0.0495 (15)	0.080 (2)	0.0454 (15)	−0.0046 (15)	0.0145 (12)	−0.0091 (14)
C17	0.0369 (13)	0.0702 (19)	0.0466 (15)	−0.0075 (12)	0.0116 (11)	−0.0066 (13)
C21	0.0384 (13)	0.0400 (13)	0.0445 (14)	0.0022 (10)	0.0065 (10)	0.0026 (11)
C22	0.0417 (14)	0.0459 (15)	0.080 (2)	−0.0006 (12)	0.0100 (14)	0.0135 (14)
C23	0.0528 (17)	0.0469 (16)	0.097 (3)	−0.0012 (13)	0.0082 (17)	0.0203 (17)
C24	0.072 (2)	0.0432 (16)	0.087 (2)	0.0092 (15)	−0.0019 (18)	0.0014 (16)
C25	0.0564 (16)	0.0471 (15)	0.0562 (17)	0.0139 (13)	0.0043 (13)	0.0021 (13)
C26	0.072 (2)	0.064 (2)	0.066 (2)	0.0321 (17)	0.0086 (16)	−0.0015 (16)
C27	0.0560 (19)	0.093 (3)	0.069 (2)	0.0343 (19)	0.0194 (16)	0.0174 (19)
C28	0.0392 (14)	0.080 (2)	0.071 (2)	0.0158 (14)	0.0134 (14)	0.0230 (17)
C29	0.0378 (13)	0.0543 (16)	0.0568 (16)	0.0100 (12)	0.0077 (12)	0.0111 (13)
C30	0.0400 (13)	0.0463 (14)	0.0461 (14)	0.0088 (11)	0.0067 (11)	0.0040 (11)
N21	0.0307 (9)	0.0397 (11)	0.0400 (11)	0.0036 (8)	0.0087 (8)	0.0016 (9)
N22	0.0303 (10)	0.0461 (12)	0.0456 (12)	0.0034 (9)	0.0115 (8)	0.0040 (9)
C31	0.0307 (11)	0.0425 (13)	0.0384 (12)	0.0028 (10)	0.0063 (9)	−0.0032 (10)
S21	0.0358 (3)	0.0414 (3)	0.0485 (4)	0.0060 (2)	0.0114 (3)	0.0056 (3)
N23	0.0335 (10)	0.0502 (12)	0.0487 (12)	0.0082 (9)	0.0098 (9)	0.0067 (10)
C32	0.0302 (12)	0.0564 (16)	0.0447 (14)	−0.0015 (11)	0.0100 (10)	−0.0048 (12)
C33	0.0385 (14)	0.083 (2)	0.068 (2)	0.0139 (15)	0.0157 (13)	0.0114 (17)
C34	0.0399 (16)	0.109 (3)	0.084 (2)	0.0103 (17)	0.0283 (16)	0.006 (2)
C35	0.0452 (16)	0.092 (2)	0.0559 (18)	−0.0123 (16)	0.0208 (13)	−0.0027 (17)
C36	0.0493 (15)	0.0673 (19)	0.0500 (16)	−0.0089 (14)	0.0131 (12)	0.0024 (14)
C37	0.0398 (14)	0.0617 (17)	0.0499 (15)	0.0004 (12)	0.0142 (11)	0.0033 (13)
S41	0.0817 (10)	0.0498 (7)	0.0839 (10)	−0.0036 (7)	−0.0225 (8)	0.0050 (7)
S41'	0.0815 (14)	0.0555 (11)	0.0591 (12)	−0.0035 (10)	0.0072 (10)	−0.0126 (9)
O41	0.0462 (10)	0.0444 (10)	0.0654 (12)	0.0011 (8)	0.0033 (9)	−0.0082 (9)
C41	0.071 (2)	0.068 (2)	0.135 (4)	−0.0099 (19)	0.010 (2)	−0.023 (2)
C42	0.067 (4)	0.062 (5)	0.238 (14)	0.028 (4)	−0.021 (8)	−0.031 (8)
C42'	0.093 (10)	0.084 (8)	0.125 (11)	0.029 (7)	0.042 (9)	−0.024 (9)

### Geometric parameters (Å, º)

**Table:**

Zn1—N1	2.057 (2)	C23—H23A	0.9900
Zn1—N21	2.064 (2)	C23—H23B	0.9900
Zn1—S21	2.2745 (6)	C24—C25	1.498 (4)
Zn1—S1	2.2747 (7)	C24—H24A	0.9900
C1—N1	1.303 (3)	C24—H24B	0.9900
C1—C10	1.471 (3)	C25—C26	1.396 (4)
C1—C2	1.503 (3)	C25—C30	1.402 (4)
C2—C3	1.529 (4)	C26—C27	1.375 (5)
C2—H2A	0.9900	C26—H26	0.9500
C2—H2B	0.9900	C27—C28	1.370 (5)
C3—C4	1.517 (4)	C27—H27	0.9500
C3—H3A	0.9900	C28—C29	1.380 (4)
C3—H3B	0.9900	C28—H28	0.9500
C4—C5	1.504 (4)	C29—C30	1.397 (4)
C4—H4A	0.9900	C29—H29	0.9500
C4—H4B	0.9900	N21—N22	1.393 (3)
C5—C6	1.387 (4)	N22—C31	1.304 (3)
C5—C10	1.403 (4)	C31—N23	1.368 (3)
C6—C7	1.380 (5)	C31—S21	1.749 (2)
C6—H6	0.9500	N23—C32	1.414 (3)
C7—C8	1.384 (5)	N23—H23N	0.8800
C7—H7	0.9500	C32—C37	1.380 (4)
C8—C9	1.386 (4)	C32—C33	1.388 (4)
C8—H8	0.9500	C33—C34	1.392 (4)
C9—C10	1.397 (4)	C33—H33	0.9500
C9—H9	0.9500	C34—C35	1.367 (5)
N1—N2	1.400 (3)	C34—H34	0.9500
N2—C11	1.303 (3)	C35—C36	1.365 (4)
C11—N3	1.369 (3)	C35—H35	0.9500
C11—S1	1.755 (2)	C36—C37	1.387 (4)
N3—C12	1.407 (3)	C36—H36	0.9500
N3—H3N	0.8800	C37—H37	0.9500
C12—C17	1.383 (4)	S41—O41	1.516 (2)
C12—C13	1.390 (3)	S41—C41	1.682 (4)
C13—C14	1.383 (4)	S41—C42	1.709 (9)
C13—H13	0.9500	S41'—O41	1.518 (3)
C14—C15	1.371 (4)	S41'—C42'	1.578 (13)
C14—H14	0.9500	S41'—C41	1.666 (4)
C15—C16	1.373 (4)	C41—H41A	0.9800
C15—H15	0.9500	C41—H41B	0.9800
C16—C17	1.394 (4)	C41—H41C	0.9800
C16—H16	0.9500	C41—H41D	0.9800
C17—H17	0.9500	C41—H41E	0.9800
C21—N21	1.303 (3)	C41—H41F	0.9800
C21—C30	1.471 (3)	C42—H42A	0.9800
C21—C22	1.499 (4)	C42—H42B	0.9800
C22—C23	1.519 (4)	C42—H42C	0.9800
C22—H22A	0.9900	C42'—H42D	0.9800
C22—H22B	0.9900	C42'—H42E	0.9800
C23—C24	1.513 (5)	C42'—H42F	0.9800
			
N1—Zn1—N21	110.30 (8)	H24A—C24—H24B	108.2
N1—Zn1—S21	132.43 (6)	C26—C25—C30	118.3 (3)
N21—Zn1—S21	87.53 (5)	C26—C25—C24	122.0 (3)
N1—Zn1—S1	88.25 (6)	C30—C25—C24	119.6 (3)
N21—Zn1—S1	129.85 (6)	C27—C26—C25	121.3 (3)
S21—Zn1—S1	114.13 (3)	C27—C26—H26	119.4
N1—C1—C10	120.3 (2)	C25—C26—H26	119.4
N1—C1—C2	120.9 (2)	C28—C27—C26	120.4 (3)
C10—C1—C2	118.7 (2)	C28—C27—H27	119.8
C1—C2—C3	113.8 (2)	C26—C27—H27	119.8
C1—C2—H2A	108.8	C27—C28—C29	119.4 (3)
C3—C2—H2A	108.8	C27—C28—H28	120.3
C1—C2—H2B	108.8	C29—C28—H28	120.3
C3—C2—H2B	108.8	C28—C29—C30	121.2 (3)
H2A—C2—H2B	107.7	C28—C29—H29	119.4
C4—C3—C2	110.2 (2)	C30—C29—H29	119.4
C4—C3—H3A	109.6	C29—C30—C25	119.0 (2)
C2—C3—H3A	109.6	C29—C30—C21	121.9 (2)
C4—C3—H3B	109.6	C25—C30—C21	119.1 (2)
C2—C3—H3B	109.6	C21—N21—N22	112.67 (19)
H3A—C3—H3B	108.1	C21—N21—Zn1	130.06 (16)
C5—C4—C3	108.8 (3)	N22—N21—Zn1	115.18 (14)
C5—C4—H4A	109.9	C31—N22—N21	116.34 (19)
C3—C4—H4A	109.9	N22—C31—N23	118.1 (2)
C5—C4—H4B	109.9	N22—C31—S21	128.12 (18)
C3—C4—H4B	109.9	N23—C31—S21	113.76 (18)
H4A—C4—H4B	108.3	C31—S21—Zn1	92.77 (8)
C6—C5—C10	118.9 (3)	C31—N23—C32	129.9 (2)
C6—C5—C4	121.8 (3)	C31—N23—H23N	115.1
C10—C5—C4	119.2 (3)	C32—N23—H23N	115.1
C7—C6—C5	121.1 (3)	C37—C32—C33	118.8 (2)
C7—C6—H6	119.4	C37—C32—N23	125.4 (2)
C5—C6—H6	119.4	C33—C32—N23	115.7 (3)
C6—C7—C8	120.3 (3)	C32—C33—C34	119.5 (3)
C6—C7—H7	119.9	C32—C33—H33	120.3
C8—C7—H7	119.9	C34—C33—H33	120.3
C7—C8—C9	119.5 (3)	C35—C34—C33	121.5 (3)
C7—C8—H8	120.3	C35—C34—H34	119.2
C9—C8—H8	120.3	C33—C34—H34	119.2
C8—C9—C10	120.6 (3)	C36—C35—C34	118.6 (3)
C8—C9—H9	119.7	C36—C35—H35	120.7
C10—C9—H9	119.7	C34—C35—H35	120.7
C9—C10—C5	119.5 (3)	C35—C36—C37	121.2 (3)
C9—C10—C1	121.9 (3)	C35—C36—H36	119.4
C5—C10—C1	118.6 (3)	C37—C36—H36	119.4
C1—N1—N2	113.40 (19)	C32—C37—C36	120.3 (3)
C1—N1—Zn1	130.03 (16)	C32—C37—H37	119.9
N2—N1—Zn1	114.81 (14)	C36—C37—H37	119.9
C11—N2—N1	116.18 (19)	O41—S41—C41	109.37 (19)
N2—C11—N3	118.5 (2)	O41—S41—C42	106.6 (4)
N2—C11—S1	128.70 (18)	C41—S41—C42	104.8 (4)
N3—C11—S1	112.77 (18)	O41—S41'—C42'	111.2 (6)
C11—S1—Zn1	92.06 (8)	O41—S41'—C41	110.1 (2)
C11—N3—C12	130.5 (2)	C42'—S41'—C41	102.6 (7)
C11—N3—H3N	114.8	S41—O41—S41'	56.91 (12)
C12—N3—H3N	114.8	S41'—C41—S41	51.17 (14)
C17—C12—C13	118.9 (2)	S41'—C41—H41A	123.9
C17—C12—N3	125.1 (2)	S41—C41—H41A	109.5
C13—C12—N3	115.9 (2)	S41'—C41—H41B	126.5
C14—C13—C12	120.5 (3)	S41—C41—H41B	109.5
C14—C13—H13	119.7	H41A—C41—H41B	109.5
C12—C13—H13	119.7	S41'—C41—H41C	58.3
C15—C14—C13	120.8 (3)	S41—C41—H41C	109.5
C15—C14—H14	119.6	H41A—C41—H41C	109.5
C13—C14—H14	119.6	H41B—C41—H41C	109.5
C14—C15—C16	118.9 (3)	S41'—C41—H41D	109.5
C14—C15—H15	120.6	S41—C41—H41D	115.9
C16—C15—H15	120.6	H41B—C41—H41D	122.4
C15—C16—C17	121.4 (3)	H41C—C41—H41D	87.2
C15—C16—H16	119.3	S41'—C41—H41E	109.5
C17—C16—H16	119.3	S41—C41—H41E	59.4
C12—C17—C16	119.5 (3)	H41A—C41—H41E	87.5
C12—C17—H17	120.2	H41B—C41—H41E	66.3
C16—C17—H17	120.2	H41C—C41—H41E	162.6
N21—C21—C30	119.9 (2)	H41D—C41—H41E	109.5
N21—C21—C22	120.9 (2)	S41'—C41—H41F	109.5
C30—C21—C22	119.1 (2)	S41—C41—H41F	134.4
C21—C22—C23	113.1 (2)	H41A—C41—H41F	114.2
C21—C22—H22A	109.0	H41C—C41—H41F	67.7
C23—C22—H22A	109.0	H41D—C41—H41F	109.5
C21—C22—H22B	109.0	H41E—C41—H41F	109.5
C23—C22—H22B	109.0	S41—C42—H42A	109.5
H22A—C22—H22B	107.8	S41—C42—H42B	109.5
C24—C23—C22	110.6 (3)	H42A—C42—H42B	109.5
C24—C23—H23A	109.5	S41—C42—H42C	109.5
C22—C23—H23A	109.5	H42A—C42—H42C	109.5
C24—C23—H23B	109.5	H42B—C42—H42C	109.5
C22—C23—H23B	109.5	S41'—C42'—H42D	109.5
H23A—C23—H23B	108.1	S41'—C42'—H42E	109.5
C25—C24—C23	109.9 (3)	H42D—C42'—H42E	109.5
C25—C24—H24A	109.7	S41'—C42'—H42F	109.5
C23—C24—H24A	109.7	H42D—C42'—H42F	109.5
C25—C24—H24B	109.7	H42E—C42'—H42F	109.5
C23—C24—H24B	109.7		
			
N1—C1—C2—C3	−176.3 (3)	C23—C24—C25—C30	33.3 (4)
C10—C1—C2—C3	1.0 (4)	C30—C25—C26—C27	−1.2 (5)
C1—C2—C3—C4	42.4 (4)	C24—C25—C26—C27	177.2 (3)
C2—C3—C4—C5	−61.7 (4)	C25—C26—C27—C28	−3.4 (5)
C3—C4—C5—C6	−138.1 (3)	C26—C27—C28—C29	3.0 (5)
C3—C4—C5—C10	38.9 (4)	C27—C28—C29—C30	2.0 (5)
C10—C5—C6—C7	−1.0 (5)	C28—C29—C30—C25	−6.6 (4)
C4—C5—C6—C7	176.0 (3)	C28—C29—C30—C21	173.8 (3)
C5—C6—C7—C8	−1.6 (6)	C26—C25—C30—C29	6.0 (4)
C6—C7—C8—C9	1.7 (6)	C24—C25—C30—C29	−172.4 (3)
C7—C8—C9—C10	0.8 (5)	C26—C25—C30—C21	−174.3 (3)
C8—C9—C10—C5	−3.4 (4)	C24—C25—C30—C21	7.2 (4)
C8—C9—C10—C1	178.3 (3)	N21—C21—C30—C29	−26.2 (4)
C6—C5—C10—C9	3.4 (4)	C22—C21—C30—C29	158.1 (3)
C4—C5—C10—C9	−173.7 (3)	N21—C21—C30—C25	154.2 (3)
C6—C5—C10—C1	−178.2 (3)	C22—C21—C30—C25	−21.5 (4)
C4—C5—C10—C1	4.7 (4)	C30—C21—N21—N22	176.8 (2)
N1—C1—C10—C9	−30.4 (4)	C22—C21—N21—N22	−7.6 (3)
C2—C1—C10—C9	152.3 (3)	C30—C21—N21—Zn1	−20.7 (4)
N1—C1—C10—C5	151.3 (3)	C22—C21—N21—Zn1	154.9 (2)
C2—C1—C10—C5	−26.0 (4)	N1—Zn1—N21—C21	−29.8 (2)
C10—C1—N1—N2	177.2 (2)	S21—Zn1—N21—C21	−164.6 (2)
C2—C1—N1—N2	−5.6 (3)	S1—Zn1—N21—C21	75.6 (2)
C10—C1—N1—Zn1	−18.9 (4)	N1—Zn1—N21—N22	132.36 (16)
C2—C1—N1—Zn1	158.3 (2)	S21—Zn1—N21—N22	−2.39 (15)
N21—Zn1—N1—C1	−32.0 (2)	S1—Zn1—N21—N22	−122.19 (14)
S21—Zn1—N1—C1	74.0 (2)	C21—N21—N22—C31	167.8 (2)
S1—Zn1—N1—C1	−164.2 (2)	Zn1—N21—N22—C31	2.5 (3)
N21—Zn1—N1—N2	131.70 (16)	N21—N22—C31—N23	178.2 (2)
S21—Zn1—N1—N2	−122.29 (14)	N21—N22—C31—S21	−1.0 (3)
S1—Zn1—N1—N2	−0.55 (15)	N22—C31—S21—Zn1	−0.7 (2)
C1—N1—N2—C11	166.6 (2)	N23—C31—S21—Zn1	−179.98 (18)
Zn1—N1—N2—C11	0.1 (3)	N1—Zn1—S21—C31	−114.08 (11)
N1—N2—C11—N3	−179.0 (2)	N21—Zn1—S21—C31	1.45 (10)
N1—N2—C11—S1	0.7 (3)	S1—Zn1—S21—C31	134.57 (8)
N2—C11—S1—Zn1	−0.9 (2)	N22—C31—N23—C32	−8.0 (4)
N3—C11—S1—Zn1	178.75 (17)	S21—C31—N23—C32	171.3 (2)
N1—Zn1—S1—C11	0.64 (10)	C31—N23—C32—C37	−0.9 (5)
N21—Zn1—S1—C11	−114.62 (11)	C31—N23—C32—C33	180.0 (3)
S21—Zn1—S1—C11	137.18 (8)	C37—C32—C33—C34	−0.5 (5)
N2—C11—N3—C12	−9.2 (4)	N23—C32—C33—C34	178.7 (3)
S1—C11—N3—C12	171.1 (2)	C32—C33—C34—C35	−0.1 (6)
C11—N3—C12—C17	−6.5 (4)	C33—C34—C35—C36	0.2 (6)
C11—N3—C12—C13	175.6 (3)	C34—C35—C36—C37	0.3 (5)
C17—C12—C13—C14	−0.4 (4)	C33—C32—C37—C36	1.0 (4)
N3—C12—C13—C14	177.7 (3)	N23—C32—C37—C36	−178.1 (3)
C12—C13—C14—C15	−0.8 (5)	C35—C36—C37—C32	−1.0 (5)
C13—C14—C15—C16	1.0 (5)	O41—S41—O41—S41'	0 (47)
C14—C15—C16—C17	−0.1 (5)	C41—S41—O41—S41'	−43.9 (2)
C13—C12—C17—C16	1.3 (4)	C42—S41—O41—S41'	68.9 (4)
N3—C12—C17—C16	−176.6 (3)	O41—S41'—O41—S41	0 (58)
C15—C16—C17—C12	−1.0 (5)	C42'—S41'—O41—S41	−68.3 (6)
N21—C21—C22—C23	177.7 (3)	C41—S41'—O41—S41	44.7 (2)
C30—C21—C22—C23	−6.7 (4)	O41—S41'—C41—S41	−42.94 (17)
C21—C22—C23—C24	46.8 (4)	C42'—S41'—C41—S41	75.5 (6)
C22—C23—C24—C25	−60.0 (4)	O41—S41—C41—S41'	42.81 (18)
C23—C24—C25—C26	−145.1 (3)	C42—S41—C41—S41'	−71.1 (5)

### Hydrogen-bond geometry (Å, º)

**Table:**

*D*—H···*A*	*D*—H	H···*A*	*D*···*A*	*D*—H···*A*
N3—H3*N*···O41	0.88	2.08	2.945 (3)	168
N23—H23*N*···O41^i^	0.88	2.03	2.903 (3)	173

Symmetry code: (i) *x*+1, *y*, *z*.
